# Transcriptome changes of disinhibited somatostatin neurons in the medial prefrontal cortex mediating male-specific stress resilience

**DOI:** 10.21203/rs.3.rs-10951363/v1

**Published:** 2026-09-09

**Authors:** Meiyu Shao, Deepro Banerjee, Anirban Paul, Santhosh Girirajan, Bernhard Lüscher

**Affiliations:** 1 Department of Biology, The Pennsylvania State University, University Park, PA; 2 Department of Biochemistry and Molecular Biology, The Pennsylvania State University, University Park, PA; 3 The Huck Institutes of the Life Sciences, The Pennsylvania State University, University Park, PA; 4 Department of Neuroscience and Experimental Therapeutics, Penn State College of Medicine, Hershey, PA; 5 Current address: Department of Stem Cell Biology and Regenerative Medicine, Keck School of Medicine, University of Southern California, Los Angeles, CA

**Keywords:** depression, anxiety, antidepressant drug action, Alzheimer’s disease

## Abstract

Somatostatin (SST)-expressing interneurons are critical modulators of vulnerability to chronic stress and are strongly implicated in the pathophysiology of stress-related affective disorders. Consistent with this role, we have recently demonstrated that selective enhancement of SST neuron activity—achieved via cell type–specific Cre-mediated inactivation of GABA_A_ receptors (SSTCre:γ2^f/f^ mice)—robustly promotes resilience to chronic variable stress (CVS) at the behavioral and tissue transcriptomic levels. Notably, the neural substrate underlying this resilience is sex-specific: in males, it localizes to the medial prefrontal cortex (mPFC), whereas in females it maps to the ventral hippocampus. Building on these findings, we sought to define the SST neuron–specific transcriptomic and biological properties associated with stress resilience in male mice. We compared purified SST neurons from the mPFC of stress-vulnerable (SSTCre) and stress-resilient (SSTCre:γ2^f/f^) male mice, using corresponding female mice as negative controls. Our analyses reveal a striking, sex-specific transcriptomic resilience phenotype. Specifically, we found that the transcriptome of mPFC SST neurons from male—but not female—SSTCre:γ2^f/f^ mice exhibits marked resistance to CVS-induced perturbations. In male SSTCre:γ2^f/f^ mice, SST neurons displayed a substantially reduced number of differentially expressed genes (DEGs) following CVS exposure, along with a loss of correlation of stress-induced transcriptional changes relative to SSTCre littermates. In contrast to males, SST neurons from the mPFC of female SSTCre:γ2^f/f^ mice exhibited an increased number of CVS-responsive DEGs and a transcriptomic profile that was inversely correlated with that of SSTCre controls. In agreement with these transcriptomic changes, gene set enrichment analyses identified markedly fewer CVS-responsive biological functions in the resilient male mice compared to their vulnerable littermates. Normalized enrichment scores for gene ontology summary terms indicated that SST neurons from male SSTCre:γ2^f/f^ mice are protected from CVS-induced downregulation of gene sets governing neural cell fate, apoptosis, and cell adhesion, and exhibit partial resilience to CVS-induced disruptions in mitogen-activated protein kinase signaling and cytoskeletal organization. By contrast, SST neurons from the female SSTCre mice showed no measurable CVS-induced alterations in these same gene programs. Collectively, these findings provide compelling evidence that the male-specific, SST neuron–intrinsic resilience mechanisms within the mPFC are key determinants of the previously described tissue-level and behavioral stress resilience phenotypes in SSTCre:γ2^f/f^ mice.

## INTRODUCTION

Chronic and excessive stress constitute major risk factors for a broad spectrum of neuropsychiatric disorders, most prominently Major Depressive Disorder (MDD) and Posttraumatic Stress Disorder (PTSD) ^1^. At the molecular level, convergent evidence from human studies and rodent models indicates that MDD, PTSD, and chronic stress exposure are associated with widespread deficits in GABAergic inhibition ^2–11^ (reviewed in ^12, 13, 14^). Complementary molecular genetic studies in mice further demonstrate that diminished GABAergic inhibition of glutamatergic neurons—particularly within the neocortex and hippocampus—is sufficient to induce anxiety- and anhedonia-like behavioral phenotypes ^15–19^. In addition, impaired GABAergic neural inhibition modulates the efficacy of antidepressant drug treatments ^17^ and contributes to downstream disruptions in glutamatergic synaptic transmission ^20^, which are likewise implicated in the pathophysiology of depressive disorders. Collectively, these findings support a causal role for reduced GABAergic function in the development of stress-associated psychiatric conditions (reviewed in ^21, 22, 23^).

Recent work has refined this framework by mapping GABAergic dysfunction to genetically and functionally distinct interneuron subtypes within the neocortex ^24^. Among these, the somatostatin (SST)-expressing interneurons are of particular interest, as they provide the principal inhibitory input to the distal dendrites of pyramidal neurons, thereby regulating cortico-cortical and thalamocortical glutamatergic integration. This function contrasts with that of the parvalbumin (Pvalb)-expressing interneurons, which target somata, proximal dendrites, and axon initial segments to exert powerful control over pyramidal neuron output. A third major interneuron subclass, defined by vasoactive intestinal peptide (VIP) expression, primarily acts to inhibit SST interneurons, thereby disinhibiting pyramidal cells.

Successful antidepressant drug treatments of patients and mice with defects in GABA function are associated with normalization of GABA concentrations and restoration of impaired GABAergic and glutamatergic synapse function ^4, 9, 20, 25–28^. These observations suggest that enhancement of GABAergic inhibition is critical for antidepressant drug action and raise the possibility that GABAergic signaling itself exerts antidepressant-like effects ^22, 29^. To directly test this hypothesis by genetic means, we recently generated mice with disinhibited GABAergic interneurons via cell type-specific, Cre-mediated ablation of the GABA_A_ receptor γ2 subunit gene (*Gabrg2)* in SST-positive interneurons (SSTCre:γ2^f/f^ mice) ^30^. We selectively targeted these neurons because SST and other markers expressed by these cells are selectively downregulated in stress- and aging-related mental disorders ^31–34^ as well as in chronic stress models of mice ^35, 36^. As predicted, SST neurons in SSTCre:γ2^f/f^ mice exhibit markedly reduced inhibitory synaptic inputs and increased intrinsic excitability, resulting in enhanced GABAergic inhibitory synaptic output onto their pyramidal cell targets in neocortex and hippocampus. At the behavioral level, these mice recapitulate key features of antidepressant drug action across multiple assays of anxiety- and depression-related behavior ^30^ (reviewed in ^22, 23^).

A defining feature of antidepressant interventions is their capacity to act prophylactically by promoting stress resilience ^37, 38^. Resilience is commonly defined as the process and outcome of successful adaptation to adversity. In rodent models of psychiatric disorders, resilience is operationalized as the absence of stress-induced alterations in anxiety- and anhedonia-like behaviors ^39–41^. Mechanistic studies in mice have predominantly relied on the chronic social defeat stress (CSDS) paradigm, which stratifies animals into susceptible and resilient subgroups based on social avoidance behavior ^39^ (for a review of major findings, see Ref. ^42^). A key feature of this approach is that it makes no prior assumptions regarding the biology underlying stress resilience. However, a significant limitation of the CSDS model is the inability to access pre-stress biological substrates that predict resilience or susceptibility.

In contrast to the CSDS model, the SSTCre:γ2^f/f^ model employed here focuses a priori on a specific GABAergic microcircuit that appears impaired in major depressive disorder ^43, 44^ and posttraumatic stress disorder ^6^, is compromised by chronic stress in mice ^32, 36, 45^ , and, when activated, promotes stress resilience ^36, 40, 41^. This model offers several advantages: i) The resilience phenotype is genetically fixed, which eliminates the need to behaviorally score every cohort of mice, ii) the genetic design provides key insights into the biology underlying resilience, and iii) the model allows for comparison of non-stressed (NS) stress-resilient (SSTCre:γ2^f/f^) mice with stressed or NS stress-susceptible (SSTCre) mice, as well as with stressed stress-resilient (SSTCre:γ2^f/f^) mice. Importantly, the model also facilitates parallel analyses in male and female mice under identical experimental conditions ^40^.

Our initial testing for resilience of the SSTCre:γ2^f/f^ strain of mice relied on the uncontrolled chronic mild stress (UCMS) protocol and revealed that male but not female mice were resilient to stress-induced anxiety ^36^. Consistent with this behavioral phenotype, phosphorylation of the eukaryotic elongation factor 2 (eEF2)—a molecular correlate of antidepressant action—was selectively attenuated in the medial prefrontal cortex (mPFC) of male mice. Subsequent studies using the chronic variable stress (CVS) paradigm demonstrated robust resilience to both anxiety- and anhedonia-like behaviors in both sexes ^40^. However, bulk tissue transcriptomic and chemogenetic analyses revealed striking sex-dependent circuit mechanisms: vulnerability and resilience mapped to the mPFC in males, but to the ventral hippocampus (vHPC) in females ^40, 41^. In the mPFC of male SSTCre:γ2^f/f^ mice, resilience was associated with a markedly attenuated whole-tissue-level transcriptional response to stress, characterized by more than a fivefold reduction in differentially expressed genes (DEGs) and a largely distinct gene expression profile compared to susceptible SSTCre littermates ^40^. Intriguingly, baseline genotype-dependent transcriptional differences between non-stressed (NS) male SSTCre:γ2^f/f^ and SSTCre mice were correlated with the transcriptional stress responses observed in SSTCre mice ^40, 46–49^. Most strikingly, CVS of SSTCre:γ2^f/f^ (resilient) mice resulted in the reversal of their stress-like transcriptome signature and was accompanied by paradoxical, anxiolytic-like behavioral effects ^40^. Therefore, increasing the baseline excitability of SST neurons in SSTCre:γ2^f/f^ mice acts like a switch, changing the normally detrimental consequences of chronic stress exposure into seemingly beneficial effects, both at the behavioral and tissue transcriptomic levels ^40^.

In the present study, we extend these findings by testing whether stress resilience is associated with sex-specific, resilience-linked transcriptomic signatures in purified SST interneurons from the mPFC.

## MATERIALS AND METHODS

### Animals

SSTCre mice (also known as Sst^tm2.1(cre)Zjh^/J, Stock No. 013044), Ai9 mice (B6.Cg-Gt(ROSA)26Sor^tm9(CAG-tdTomato)Hze^/J, Stock No.007909) carrying the Cre-dependent fluorescent marker tdTomato (tdT), and C57BL/6J mice (B6, Stock No. 000664) were all obtained from Jackson Laboratory (Bar Harbor, ME, USA). The γ2^f/f^ mouse line (Gabrg2^tm2Lusc^/J, Stock No: 016830, Jackson Laboratory) containing a *Gabrg2* allele flanked by loxP sites was generated in-house ^50^. All mice were backcrossed to the B6 strain for at least five generations and maintained on corn cob bedding on a 12:12 h light-dark cycle with food and water available ad libitum. To avoid interference with analyses by a hypomorphic Cre allele, the SSTCre allele was strictly maintained in the heterozygous state. The mice compared experimentally were produced by crossing SSTCre:γ2^f/+^:tdT/tdT male mice with γ2^f/+^ female mice or by crossing SSTCre:γ2^f/+^ male mice with γ2^f/+^:tdT/tdT female mice. The mice were genotyped by PCR of tail biopsies at the time of weaning using primers previously described ^50–52^. Equal groups of SSTCre:tdT and SSTCre:γ2^f/f^:tdT littermates were subjected to CVS or no stress (NS) control conditions, with male and female mice analyzed sequentially from separate cohorts for practical reasons (see Figure 1 for an illustration of the experimental design). Given the presence of the tdT marker in all the mice, it is not further mentioned in the Results and Discussion for simplicity. All animal experiments were performed following the guidelines of the National Institutes of Health (NIH) and approved by the Institutional Animal Care and Use Committees (IACUC, PRAMS200346483 and PRAMS201745805) of The Pennsylvania State University.

### Chronic Variable Stress (CVS) Exposure

The experimental groups of mice, which differed by sex and genotype, were further divided into NS and CVS groups, balancing for sucrose preference and body weight, and housed 2–3 per cage. CVS was initiated at 8–10 weeks of age and performed as previously described ^40^. Briefly, the procedure included three different stressors repeated for a total of 21 days, starting on day one with a one-hour tail suspension stressor, followed on day two with a one-hour restraint stressor, and on day three with exposure to 100 randomly distributed foot shocks (0.45 mA * 3 s) within one hour. After each stressor, the mice were returned to their home cage. The brains of CVS-exposed mice were harvested and processed 24–28 h after the final stressor. NS controls were group-housed in a separate room under standard housing conditions and handled every other day. The harvest order of the four types of mice—NS SSTCre:tdT, CVS SSTCre:tdT, NS SSTCre:γ2^f/f^:tdT, and CVS SSTCre:γ2^f/f^:tdT mice—was rotated over 4 days to ensure that the last stressor before tissue isolation was the same for all the mice. This experimental design prevented blinding of the investigators to genotype and stress treatment. In each sample, the mPFC brain slices from 1–2 transgenic mice were enzymatically dissociated, stained with the cell viability stain, calcein, and sorted to purify tdT^+^/calcein^+^ live neurons (Figure 1 **and S1**). The purified cells were frozen in RNA extraction buffer at −80 °C for further processing after the collection of all samples was complete. Total RNA from the purified samples was then used for cDNA library construction and subjected to RNA-seq. The sample size (n = 4) was chosen based on previous RNA-Seq bulk tissue analyses of NS and CVS-exposed SSTCre:tdT and SSTCre:γ2^f/f^:tdT mice ^40^. Some mixed cell samples representing the recombined fractions of cells after FACS were processed in parallel to assess the degree of enrichment and depletion of different cell types in the purified samples.

### Fluorescence-Activated Cell Sorting (FACS)

The mice (one at the time) were anesthetized with 30 ml/kg of 1.25% Avertin (12.5 g/L of 2.5% 2,2,2-tribromoethanol, 5 mL 2-methyl-2-butanol), perfused with pre-chilled oxygenated artificial cerebrospinal fluid (ACSF, in mM: NaCl 124, NaHCO_3_ 26 2, glucose 10, MgSO_4_ 1.3, NaH_2_PO_4_ 1.25, KCl 2.5, CaCl_2_ 2.5, Na L-ascorbate 1.3, Na pyruvate 0.6, pH 7.4). The brains were cut into 1-mm wide coronal sections using a brain matrix (Stoelting Co., Wood Dale, IL), and the sections were transferred to pre-chilled DPBS (in mM: KCl 2.68, KH_2_PO4 1.47, NaCl 137, Na_2_HPO_4_ 25.2, glucose 5.6, Na-pyruvate 0.327, Mg_2_Cl 0.49, Ca_2_Cl 0.9, pH 7.4) containing 30 μM actinomycin D (ActD). The expression of tdT was confirmed under a fluorescent microscope using an olfactory bulb smear. The mPFC (Cg1, PrL, and IL ^53^) was dissected, minced, and digested for 30 min at 37°C in 1.5 mL of an enzyme mix (Adult Brain Dissociation Kit mouse and rat, Miltenyi Biotec, Waltham, MA) supplemented with 30 μM ActD. The reaction was terminated by addition of 500 μL of 0.5% bovine serum albumin in DPBS. The tissue was triturated using a series of polished Pasteur pipettes with declining diameters of 600 μm, 300 μm, and 150 μm, and the homogenate was passed through a 70 μm filter and washed twice in DPBS supplemented with 0.5% bovine serum albumin (BSA, Research Product International, Mount Prospect, IL) by resuspension and centrifugation. The cell pellet was resuspended in 500 μL DPBS/0.5% BSA/2 mM ethylene diamine tetraacetate (EDTA), filtered through a 35 μm Falcon filter (Corning, Pittsburgh, PA), and stained with 250 nM Calcein Violet (CellTrace^™^, Invitrogen) for 15 min on ice. The Calcein Violet, tdT doubly positive cells were sorted on a MoFlo Astrios EQ Cell Sorter (Beckman Coulter Life Sciences) using a 100 μm nozzle at 25 psi (**Figure S1A-H**). The gating parameters were optimized empirically through microscopy and scoring of particle size and shape, and by the absence of aggregates. Ultimately, we assessed the purity of samples using PCR of reverse-transcribed RNA and by pilot RNA-Seq reactions and testing for marker gene enrichment and depletion. In particular, RNAseq of mixed samples consisting of the recombined fractions of sorted samples was used to quantify enrichment and depletion of marker gene transcripts in purified samples sequenced in parallel (**Figure S1I)**. The sorting of samples by forward scatter (FSC) alone was inadequate due to the wide range of sizes and shapes of singlets and debris. Side scatter (SSC) allowed for a more efficient separation of viable single cells from cell debris and aggregates. Accordingly, live single cells were selected using sequential SSC-area/FSC-area, SSC height/SSC, and FSC area/FSC height plots, followed by selection of cells based on tdT and calcein fluorescence (**Figure S1A–F**). The yield of calcein-tdT double-positive cells ranged between 2,000 and 4,000 cells per sample. For RNA-seq, the cells were collected in 100 μL RNA extraction buffer (PicoPure RNA isolation kit, ThermoFisher Scientific), incubated at 42°C for 30 min, and stored at −80°C until all samples were available for further processing.

### Validation of FACS-Purified SST Cells

Inspection of purified SST neuron samples by fluorescent microscopy showed tdT expression in virtually all the cells (**Figure S1G**). PCA analyses of RNA-seq data revealed a clear separation of purified neuron samples from total mixed cell samples (**Figure S1H**). By contrast, the samples from male and female mice were all co-clustered, indicating that their transcriptomes are similar irrespective of genotype and CVS exposure. Comparison of RNA-seq read counts for cell-specific marker genes between purified SST neuron samples and mixed samples (prepared by recombining the fractions of dissociated cells after FACS) showed that markers for endothelial cells (Flt1, Nos3), oligodendrocytes (Pdgfra, Cspg4), astrocytes (*Sox9*, *Aqp4*, *Gja1*), and microglia (*Ctss*, *Itgam*, *Cx3cr1*, *Gpr34*) were all strongly depleted (**Figure S1I**). Similarly, the transcripts for the vesicular glutamate transporters *SLC17a6–8* (coding for VGLUT1–3), representative of glutamatergic neurons, were reduced, reflecting an average 227-fold reduction in the ratio of total *SLC17a6–8* vs. *Sst* transcripts in purified vs. mixed samples. By contrast, markers expressed across GABAergic neurons (*Slc32a1*, *Gad2*, *Gad1*) were enriched, as expected. Similarly, marker genes known to be expressed in SST neurons, including *Sst* (fold enrichment, 73.4), *Chodl* (122.3), *Oxtr* (16.5), *Npy* (14.0), *Hpse* (10.6), *Nos1* (5.9), and *Calb2* (5.5), were highly enriched. *Pvalb* was also modestly enriched (about two-fold), in keeping with single-cell data indicating that most SST cell types express low levels of *Pvalb* ^54^ and low levels of overlap between *SST*- and *Pvalb*-expressing neurons ^55^.

### RNA Sequencing (RNA-seq), Code Availability

Total RNA was extracted from the sorted cells using a PicoPure RNA isolation kit (ThermoFisher Scientific) with an on-column DNase I digestion step (Qiagen). The samples were converted into libraries using a SMART-Seq^®^ Stranded Kit with an rRNA depletion step using scZapR (TakaRa). The RNAs were fragmented for four minutes as described by the kit manufacturer, followed by first-strand cDNA synthesis, addition of Illumina dualindex adapters using eight polymerase chain reaction (PCR) cycles, ribosomal RNA digestion, and amplification for an additional 10 cycles. The libraries were purified using an AMPure XP PCR purification kit (Beckman Coulter) and then sent to Novogene Corporation for sequencing on an Illumina NovaSeq 6000 system to a depth of 25 million 150-bp paired-end reads per sample. The sequencing adapters and reads < 50 bp were removed using fastp v.0.23.2 ^56^, and the reads were aligned to GRCm39 using STAR aligner v.2.7.10b ^57^. The STAR genome index was constructed using the annotation v.M32 downloaded from GENCODE. FeatureCounts ^58^ was utilized to quantify read pairs aligned to exons at the gene level in a strand-specific mode. Read pairs that aligned to multiple genes were excluded from the analysis. The sequence data can be accessed from NCBI (GSE330271). The code for RNA-seq data analysis is available from the corresponding author upon request.

### Differential Expression Analyses

The mRNA read pair counts were extracted from RNA-seq data. DEGs were identified using DESeq2 v.1.40.2 ^59^. Genes expressed at low levels were filtered out if the sum of read pairs across all samples was less than the number of samples in the smallest condition group. A cutoff of p < 0.01 was used to define DEGs, except as indicated otherwise. The lists of DEGs are available in Tables S1–S6.

### Batch Normalization and Principal Component Analyses

Principal Component Analyses (PCA) were conducted using the DESeq2 R package ^59^. Batch differences between male and female samples were removed using ComBat-seq ^60^, followed by a variance-stabilizing transformation (vst ()). The top 1,000 genes based on variance were used for PCA using the plotPCA () function.

### Rank-Rank Hypergeometric Overlap (RRHO) Analyses

We used RRHO analyses as a threshold-free approach to compare and visualize global patterns of transcriptional regulation between pairs of differentially expressed gene sets. We prepared gene lists containing all overlapping genes between two comparisons and ranked the genes by [−log10(p-value) * sign(log2FC)]. Plots of heatmaps comparing pairs of gene sets were generated in R using the RRHO2 package ^61^ using default settings (github.com/RRHO2/RRHO2). The four quadrants of RRHO plots represent genes upregulated under the two conditions compared (bottom left), genes upregulated under one condition and downregulated under the other (top left and bottom right), and genes downregulated under both conditions (top right), while heat indicates −log10(p-value) as a measure of the strength of concordance and discordance of gene expression ^61^. We employed the same maximal scale of 1 to 1200 for −log_10_(*p*-value) for RRHO plots to facilitate comparison of heatmaps between datasets. Depiction of the RRHO plots at a smaller scale of 1 to 500 did not uncover any correlations not evident at the larger scale (data not shown).

### Gene Set Enrichment Analyses (GSEA)

The expression data of all genes were ranked based on log2 fold changes (FC) and used as input for Gene Set Enrichment Analysis (GSEA) ^62^. The ‘gseGO ()’ function from the clusterProfiler R package ^63^ was used to identify significantly altered GO terms using a cutoff of p_adj_ < 0.05. GO summary terms were selected from the GSEA results using keyword-based filtering with a focus on biological processes known to be affected by stress. Specifically, GO summary terms related to neural cell fate were identified using the keywords “neuron” and “nervous”; translation using “translation”; apoptosis and programmed cell death (PCD) using “apop” and “death”; cytoskeleton using “actin”, “filament” and “skeleton”; mitogen-activate protein kinase (MAPK) signaling using “MAPK” and “stress”; cell adhesion using “adhesion”, and synapse function using “synapse”. GO summary terms related to endoplasmic reticulum (ER) stress and the unfolded protein response (UPR) were searched using the terms listed in **Figure S2H** and retrieved from Ref ^64^. To avoid redundancy, each GO term was included only once in the final list. The normalized enrichment scores (NES) of GO terms were automatically computed by the R package clusterProfiler, available on the GSEA website (https://www.gsea-msigdb.org/gsea/index.jsp ^65^). The lists of GSEA results are available in Tables S7–S12.

### Weighted Gene Correlation Network Analyses (WGCNA)

Genes with low expression were filtered out if the sum of read pairs across all samples was less than the number of samples in the smallest condition group. vst() function from DESeq2 R package was used to generate variance-stabilized expression counts for each gene. The top 5,000 most variable genes, ranked by median absolute deviation, were selected for network construction using WGCNA v.1.74 ^66^. The soft-thresholding power was determined using a scale-free topology criterion, with the fit index (R^2^) cutoff set to 0.80. Co-expression networks were constructed separately for males and females using the following parameters: signed network, signed topological overlap matrix, Pearson correlation, module size (30–5000), and merge cut height (0.35). Genes from modules (MEblack in males and MEblue in females) showing significant stress x genotype effects were selected for Gene Ontology (GO) enrichment analysis using clusterProfiler v. 4.18.4 R package. For the MEblack module in males and the MEblue module in females, intramodular connectivity was calculated, and the top 50 hub genes were selected. Networks were exported to Cytoscape v3.10.4 for visualization, with edge weight thresholds of 0.12 (males) and 0.45 (females), where the higher threshold in females was applied to reduce network density.

### Statistics

Testing for stress x genotype interactions among eigengene group means employed two-way ANOVAs followed by Tukey’s post hoc test, using Prism 10 software (GraphPad, La Jolla, CA). All other statistical analyses were integral to the bioinformatic and R packages detailed in the respective Methods earlier.

## RESULTS

### SST neurons from the mPFC of male SSTCre:γ2^f/f^ mice exhibit an attenuated, qualitatively distinct stress response indicative of stress resilience.

We previously demonstrated that SSTCre:γ2^f/f^ mice are resilient to CVS-induced changes in anxiety- and anhedonia-like motivated behavior, independent of sex ^40^. Behavioral resilience was associated with resilience to CVS-induced bulk tissue transcriptome changes in the mPFC of male but not female mice ^40^. These findings confirmed prior chemogenetic studies indicating that the neural substrates underlying SST neuron-mediated resilience are sex-specific ^41^. To test whether the behavioral and tissue-level resilience of male SSTCre:γ2^f/f^ mice is driven by intrinsic resilience of SST neurons, we first compared the CVS-induced DEGs from SST neurons from the mPFC of SSTCre (vulnerable) mice with those from SSTCre:γ2^f/f^ (resilient) mice (all animals contained the tdT marker, which is omitted for clarity). Using a stringent threshold (p_adj_ < 0.05) as a cutoff for DEGs, CVS exposure resulted in 72 DEGs in SST neurons from SSTCre mice, whereas none were detected in SST neurons from SSTCre:γ2^f/f^ mice (Figure 2A, B), indicating marked transcriptomic resilience in SST neurons from male SSTCre:γ2^f/f^ mice. However, under the same conditions, no DEGs were detected in female mice (not shown). For a more comprehensive examination of the findings, we reanalyzed the data using a more relaxed cutoff (p < 0.01). CVS resulted in 456 DEGs in SST neurons from SSTCre male mice, compared to 308 CVS-induced DEGs in SSTCre:γ2^f/f^ male mice (Figure 2A, B). The attenuated transcriptional response to CVS in SST neurons from SSTCre:γ2^f/f^ mice relative to SSTCre controls indicates that disinhibition of SST neurons confers cell-autonomous stress resilience.

Previous studies based on the CSDS model have demonstrated that stress resilience is not just a passive state but rather an active process characterized by stress-induced gene expression programs that are absent in vulnerable individuals ^39, 67^. Accordingly, if SST neurons in male SSTCre:γ2^f/f^ mice exhibit properties of stress resilience, their residual CVS-induced transcriptome changes should be qualitatively distinct from those observed in vulnerable SSTCre littermate controls. Consistent with this prediction, comparison of CVS-responsive transcriptomes revealed minimal overlap between the two genotypes, with only ~1% shared DEGs (Figure 2C). Moreover, the directionality of gene expression changes differed strikingly between strains. In SSTCre (vulnerable) mice, the vast majority of DEGs (391 of 456) were downregulated following stress. In contrast, DEGs in SST neurons from SSTCre:γ2^f/f^ (resilient) mice were more evenly distributed between up- and downregulated genes (159 and 149 of 308, respectively; Figure 2D, E). Specifically, genes altered by CVS in SSTCre mice (Figure 2D, top row) exhibited no consistent directional pattern in SSTCre:γ2^f/f^ mice (bottom rows), appearing instead randomly regulated. The converse was also true: DEGs identified in SSTCre:γ2^f/f^ mice (Figure 2E, top row) showed no coherent directional response in SSTCre controls (bottom row). Together, these findings indicate that CVS elicits fundamentally distinct transcriptional programs in the two genotypes.

To extend these observations beyond DEG-level comparisons, we performed RRHO analyses across the full transcriptome. In agreement with the DEG results, RRHO plots revealed no detectable overlap between genotypes, indicating a lack of both positive and negative correlation in their stress responses (Figure 2F). Collectively, these data support the conclusion that SST neurons in the mPFC of male SSTCre:γ2^f/f^ mice exhibit a resilient transcriptional phenotype, characterized not only by fewer DEGs but also by gene expression changes that are distinct from—and uncorrelated with—those observed in vulnerable controls.

### SST neurons from the mPFC of female SSTCre:γ2^f/f^ mice lack signs of resilience and instead exhibit an exacerbated stress response.

We previously reported that the mPFC bulk transcriptome of female SSTCre:γ2^f/f^ mice lacks the signatures of stress resilience observed in males ^40^. This finding is consistent with chemogenetic studies demonstrating that SST neuron-mediated behavioral resilience in females is mediated by the ventral hippocampus rather than the mPFC ^41^. Consequently, the transcriptomes of mPFC SST neurons purified from female CVS-exposed SSTCre:γ2^f/f^ compared with SSTCre mice are not informative regarding mechanisms of resilience. However, the data from female mice can serve as a litmus test for the significance of the transcriptome changes in SST neurons from male mice. If SST neurons in the female mice showed CVS-responsive transcriptome signatures similar to those in males, such a finding would argue against a role for the male transcriptomic signatures in resilience.

Strikingly, the female data diverged sharply from those of males. CVS resulted in substantially more DEGs in SST neurons from SSTCre:γ2^f/f^ females (633) than in SSTCre controls (226; Figure 2G–I), indicating an exacerbated rather than buffered stress response. In addition, the DEGs of the two strains of female mice showed six times greater (6% vs. 1%) overlap than in the males (Figure 2I vs. C). Furthermore, the heatmaps indicated that the CVS-responsive DEGs in SST cells from the female SSTCre mice were mostly upregulated by CVS and directionally opposite to those in SSTCre:γ2^f/f^ mice (Figure 2G, J, K), whereas in the males, the CVS-responsive DEGs in SSTCre and SSTCre:γ2^f/f^ mice were directionally uncorrelated (Figure 2D, E). Lastly, RRHO plots comparing the transcriptome-wide CVS responses in SST cells from the female SSTCre:γ2^f/f^ and SSTCre mice revealed opposite (also referred to as discordant or anticorrelated ^61^) changes in expression (Figure 2L), in contrast to the uncorrelated relationship in the males (Figure 2F). Altogether, and consistent with our previously described tissue-level transcriptomes ^40^, the transcriptome of SST cells in the mPFC of CVS-exposed female SSTCre:γ2^f/f^ mice lacked features of resilience, reinforcing the conclusion above that the mPFC SST neuron transcriptome of CVS-exposed male SSTCre:γ2^f/f^ mice provides a mechanistic foundation for stress resilience.

### The transcriptome of SST cells from stress-exposed stress-vulnerable mice resembles that of stress-naive resilient mice, independent of sex.

Comparison of CVS-induced DEGs in SST neurons from SSTCre male mice with genotype-induced DEGs (NS SSTCre:γ2^f/f^ vs. NS SSTCre mice) revealed a substantial overlap. About one third (148 of 456) of the CVS-induced DEGs in SSTCre mice were also identified in the genotype comparison (Figure 3A). RRHO of CVS responses in SST neurons from SSTCre mice with genotype-induced changes showed significant concordance of gene expression changes between the two contrasts (Figure 3B). Notably, the concordance was much greater for genes downregulated under both conditions (upper right quadrant of the RRHO plot in Figure 3B) than for those upregulated under both conditions (lower left quadrant of the plot). This concordance between the stress-induced transcriptome of SST neurons from stress-vulnerable mice and the transcriptome of SST neurons from stress-naïve resilient mice indicates that SST neurons from the stress-naïve resilient mice exhibit features of stress exposure and, by extension, that some of the stress-induced transcriptional changes observed in vulnerable mice may be naturally protective (see Discussion).

We next extended the same analyses to female mice as an additional reference. Venn diagram analysis indicated that approximately two-thirds (150 of 226) of CVS-induced DEGs in SST cells from SSTCre mice overlapped with genotype-induced DEGs (NS SSTCre:γ2^f/f^ vs. NS SSTCre) (Figure 3C). Notably, SST cells from NS female mice showed an overtly larger number of genotype-induced DEGs than those from the males (Figure 3A, C), indicating that disinhibition of SST neurons had a greater impact on the SST cell transcriptome in females than in males (see also Figure 4E, below, and the Discussion). RRHO analysis of CVS-induced transcriptional changes in SST neurons from SSTCre females and genotype-induced transcriptional changes (NS SSTCre:γ2^f/f^ vs. NS SSTCre) showed a high level of concordance, similar to but more robust than in males. Collectively, these data indicate that the transcriptional responses to SST neuron disinhibition are greater than the CVS effects on SST neurons from SSTCre mice and that they are qualitatively similar in male and female mice, as we confirmed by direct comparison further below.

### Chronic stress reverses the stress-like transcriptomic signature in SST neurons of resilient mice.

We recently showed that the tissue-level transcriptional changes in the mPFC of male NS SSTCre:γ2^f/f^ vs. NS SSTCre mice were significantly anticorrelated with transcriptional changes in CVS-exposed SSTCre:γ2^f/f^ vs. NS SSTCre:γ2^f/f^ mice ^40^. In other words, the stress-like transcriptome signature of the stress-naïve resilient male mice (NS SSTCre:γ2^f/f^ vs. NS SSTCre) was reversed and partly normalized upon stress exposure. Behaviorally, this reversal is associated with paradoxical anxiolytic-like behavioral effects, suggesting that stress-induced transcriptomic reprogramming may confer therapeutic-like beneficial effects ^40^. Here, we asked whether similar patterns are evident in purified SST neurons from male mice. Venn diagram analysis revealed a greater number of DEGs in SST neurons from male NS SSTCre:γ2^f/f^ vs. NS SSTCre mice than in CVS vs. NS SSTCre:γ2^f/f^ mice, with little overlap (Figure 3E). RRHO of the two contrasts revealed significantly anticorrelated (opposite) changes (Figure 3F). Therefore, these results closely mirror prior observations at the tissue level ^40^, supporting the conclusion that activity- and stress-dependent transcriptional programs in SST neurons drive corresponding changes in bulk tissue transcriptomes.

Parallel analyses in female mice yielded comparable results. Genotype-induced DEGs (NS SSTCre:γ2^f/f^ vs. NS SSTCre) again outnumbered CVS-induced DEGs (Figure 3G), and RRHO analysis revealed a similarly strong anticorrelated relationship between genotype- and stress-induced transcriptional changes (Figure 3H). Therefore, the genetically induced changes that lead to stress resilience in SST neurons of the male mice are conserved between the sexes and even more robust in females vs. males. This finding is expected since the genetic manipulation of SST neurons in male and female SSTCre:γ2^f/f^ mice is identical. Moreover, the impact of the genetically induced disinhibition appears to have a greater impact in females than in males due to the previously mentioned lower baseline excitability of SST neurons in females than in males ^68^ (see also the Discussion). It follows that sex differences in SST neuron-mediated resilience are due to sex differences in stress-induced neuronal vulnerability rather than sex-specific effects of SST neuron disinhibition.

### Sex differences in the SST neuron transcriptome associated with stress resilience of SSTCre:γ2^f/f^ mice.

We previously showed that direct chemogenetic chronic stimulation of prelimbic SST neurons conferred resilience in chronic stress-induced anxiety- and anhedonia-like behavior in male but not female mice ^41^. Consistent with this, the mPFC bulk tissue transcriptome of SSTCre:γ2^f/f^ mice showed evidence of stress resilience in male but not female mice ^40^. To directly compare the transcriptomes of SST cells from male and female mice, we batch-normalized the corresponding data sets. Venn diagram comparisons of the CVS-induced DEGs in SST neurons purified from the mPFC of male and female SSTCre (vulnerable) mice revealed negligible overlap (1%), indicating divergent transcriptional responses (Figure 4A). Consistent with this, RRHO analyses of transcriptome-wide CVS-induced transcriptional changes showed neither concordant nor discordant regulation by CVS (Figure 4B), confirming that stress induces fundamentally distinct transcriptional programs in SST neurons from male and female mice.

Next, we compared the CVS responses of SST cells from male and female SSTCre:γ2^f/f^ (resilient) mice. Although we again found negligible overlap between the DEGs (Figure 4C), the CVS-induced transcriptomes of male and female SSTCre:γ2^f/f^ mice showed weak concordance (Figure 4D), suggesting that disinhibition of SST neurons reduced the sex differences observed in SSTCre mice (Figure 4B). These findings indicate that genetic disinhibition dominates the functional properties of SST neurons and overrides the known sex differences in intrinsic excitability of mPFC SST neurons in SSTCre mice ^68^.

Analogous comparison of genotype-induced DEGs of mPFC SST cells (NS SSTCre:γ2^f/f^ vs. NS SSTCre) between male and female mice revealed more substantial overlap, with more than a third of the DEGs seen in males (379 of 1042, 36%) also observed in females (Figure 4E). By contrast, the large majority (2012 of 2481, 81%) of DEGs observed in females were female-specific. This suggests that disinhibition of SST cells has similar effects on the transcriptome in male and female mice. However, the effects are more robust in females than in males, most likely due to the lower basal excitability of SST neurons in females than in males ^68^, which suggests a larger relative effect of disinhibition in females than in males. A transcriptome-wide comparison by RRHO revealed robust concordance (Figure 4F), confirming the qualitatively similar effect of SST disinhibition in male and female mice.

### Disinhibition of SST neurons results in sex-specific resilience to stress-induced gene set changes in SST neurons.

To assess the functional changes associated with resilience in SST neurons, we first performed GSEA. Similar to RRHO, GSEA considers the entire transcriptome to determine predefined gene sets representing biological terms and pathways (also known as Gene Ontology (GO) terms) with concordant gene expression changes within a group of mice. A Venn diagram comparing the CVS-induced changes in mPFC SST cells indicated that 1780 gene sets (p_adj_ < 0.05) were significantly altered by CVS in SST cells of male SSTCre (vulnerable) mice, while fewer than half as many (630) were affected by CVS in the SSTCre:γ2^f/f^ (resilient) strain, and 215 gene sets were changed in both types of mice (Figure 5A). Both the smaller number of CVS-responsive gene sets and the limited overlap are consistent with SST cells in the mPFC of male SSTCre:γ2^f/f^ mice exhibiting stress resilience. An analogous analysis of SST cells from female mice revealed a mere 50 gene sets affected by CVS in SST cells from SSTCre (vulnerable) mice and a much larger number of CVS-responsive gene sets (299) in SSTCre:γ2^f/f^ (resilient) mice (Figure 5B). These data confirm that SST cells from the mPFC of female SSTCre mice are less vulnerable to CVS than those from males, which is consistent with our previous observation that tissue-level vulnerability and resilience in female SSTCre:γ2^f/f^ mice map to the vHPC and not the mPFC ^41^. In addition, the CVS-induced gene set changes in the female SSTCre:γ2^f/f^ mice were anticorrelated with those in the SSTCre mice, which aligns with the anticorrelated DEGs and transcriptomes of the two strains of mice (Figure 2J–L) (see the Discussion for an explanation of this female-specific stress effect).

To assess the functional changes in SST cells associated with stress resilience, we next performed correlational analyses of normalized enrichment scores (NES, p_adj_ < 0.05) for the CVS-regulated gene sets that overlapped between the mice compared. A positive NES value indicates that the genes annotated to the corresponding GO term are predominantly found at the top of a list of genes ranked based on altered expression and, therefore, that the gene set is enriched for genes whose transcripts are upregulated. Conversely, a negative NES indicates that the majority of the genes annotated to the corresponding GO term are near the bottom of the ranked list of genes and downregulated. Interestingly, the NES for the CVS-induced GO terms that overlapped between SST neurons from SSTCre (vulnerable) and SSTCre:γ2^f/f^ (resilient) male mice were virtually all negative, indicating the gene sets were predominantly downregulated. Although they were affected in the same direction, they were uncorrelated between the two genotypes (r = 0.12**,** Figure 5A. By contrast, the NES for the CVS-induced GO terms that overlapped between females of the two mouse strains was strongly anticorrelated (r = −0.94) (Figure 5B), indicating that the overlapping CVS-regulated gene sets of the two mouse strains were affected in opposite directions. Lastly, comparative GSEA of CVS-induced GO terms from SSTCre (vulnerable) male mice with genotype-induced GO terms (NS SSTCre:γ2^f/f^ vs. SSTCre male mice) showed a prominent overlap with almost half of the CVS-induced GO terms of SSTCre mice (828 of 1780) also affected by the genotype comparison, and the NES for the corresponding overlapping gene sets were positively correlated (r = 0.4) (Figure 5C). While most of the gene sets were downregulated in both contrasts, as indicated by negative NES values, a few gene sets were prominently upregulated in both contrasts. The positive correlation of NES remained intact after we deleted the upregulated GO terms from the dataset (r = 0.42, data not shown), indicating that the correlation was not merely driven by the upregulated gene sets. The data indicate that disinhibition-induced functional changes observed in SST neurons derived from NS resilient mice mimic the CVS-induced functional changes in SST neurons from vulnerable mice, a feature we had previously observed also at the level of the corresponding tissue transcriptomes ^40^ (see Discussion).

### Stress-resilience of SST neurons from male SSTCre:γ2^f/f^ mice involves attenuation of stress-responsive gene sets implicated in stress vulnerability, as well as enhanced mRNA translation.

To evaluate the biological changes associated with resilience of SST cells, we focused on GO terms a priori tied to stress exposure such as neuronal cell fate ^69, 70^, apoptosis and PCD ^71^, cell adhesion ^72^, MAPK signaling ^73, 74^, the cytoskeleton ^75, 76^, mRNA translation ^36, 37^, ER stress/UPR ^45, 64^, and synapse function ^77^. We extracted lists of GO terms from the total number of significantly affected gene sets (p_adj_ < 0.05) for each of these terms and compared heat maps of their normalized enrichment scores (NES, p_adj_ < 0.05) for SST cells from male CVS-exposed SSTCre (vulnerable) mice, from male CVS-exposed SSTCre:γ2^f/f^ (resilient) mice, and for the genotype comparison of male NS SSTCre and NS SSTCre:γ2^f/f^ mice, as well as for these same contrasts from female mice as additional controls. The summary GO terms for neuronal cell fate, apoptosis/PCD, adhesion, MAPK, and the cytoskeleton all showed negative NES values for SST neurons from CVS vs. NS SSTCre male mice, indicating that these functions were downregulated by stress and suggesting that they contributed to stress vulnerability. Moreover, these same effects were partly (MAPK, cytoskeleton) or completely (neural cell fate, apoptosis/PCD, adhesion) attenuated in CVS vs. NS SSTCre:γ2^f/f^ male mice, suggesting a contribution to stress resilience (**Figure S2A-E**) (summarized in Table 1). The gene sets for apoptosis/PCD and adhesion also showed comparable negative NES for the genotype comparison of NS male mice (**Figure S2B, C**), suggesting that their downregulation primes SST neurons of SSTCre:γ2^f/f^ male mice for resilience even before the mice experience stress. Interestingly, the NES values for translation-related GO terms were all negative for SST cells from CVS-exposed SSTCre:γ2^f/f^ (resilient) male mice (**Figure S2F**), indicating that translation is downregulated in SST neurons from CVS-exposed SSTCre:γ2^f/f^ male mice, a phenotype that is opposite to what we had observed previously at the bulk tissue level ^40^. Nevertheless, translation-related GO terms were increased in SST neurons of CVS-exposed stress-resilient mice (**Figure S2F**), indicating that stress exposure of SSTCre:γ2^f/f^ mice normalized the expression of these gene sets. All these gene set changes were specifically observed in male but not female mice, which is consistent with the premise that resilience-related mechanisms must be male-specific.

The synapse-related gene sets in SST neurons from SSTCre (vulnerable) mice were largely unaffected by stress. Although these gene sets were up-regulated in NS SSTCre:γ2^f/f^ vs. NS SSTCre mice and downregulated by stress in SSTCre:γ2^f/f^ mice **(Figure S2G)** (Table 1) these changes did not compensate for a stress vulnerability of synapses, suggesting they were unlikely to contribute to resilience. Surprisingly, none of the ER stress/UPR-related gene sets previously implicated in vulnerability of SST neurons (**Figure S2H**) were significantly enriched. Possible reasons for this apparent discrepancy with the literature are discussed.

As a litmus test to further examine the different GO summary terms for stress resilience, we performed the same analyses in female mice (**Figure S2A–H**, summarized in Table 1). Because the substrate of stress resilience in female SSTCre:γ2^f/f^ mice maps to the vHPC rather than the mPFC, it follows that enrichment of corresponding CVS-responsive gene sets in the female mPFC is unrelated to resilience of these mice and not informative. By extension, a gene set that is similarly enriched in females and males fails the litmus test of male specificity and is unlikely to contribute to resilience in the males. Interestingly, we found that none of the cell fate-, apoptosis/programmed cell death-, adhesion-, MAPK-, cytoskeleton-, and translation-associated gene sets were significantly affected by CVS in SST cells from female SSTCre mice, and they were largely unaffected by CVS in SST cells from the SSTCre:γ2^f/f^ (resilient) strain. The male-specific nature of the above CVS-induced changes in SSTCre mice substantiates their relevance for stress resilience in SST neurons of male SSTCre:γ2^f/f^ mice. By contrast, the synapse-related changes in NES values were comparable in male and female mice, indicating that they failed the litmus test and do not contribute to the male-specific resilience mechanism.

In an attempt to corroborate the above findings from GSEA, we used WGCNA to identify co-expressed gene clusters (modules) and module-associated GO summary terms implicated in resilience. WGCNA of RNAseq data from males identified nine co-expression gene modules that were differentially depleted or enriched in SST neurons across the four different groups of mice (**Figure S3A)**. We predicted that modules contributing to resilience would show opposite CVS effects in SST neurons from SSTCre:γ2^f/f^ vs. SSTCre mice. Two-way ANOVAs revealed a single module (MEblack) with a significant interaction between stress and genotype of mean eigengene values (**Figure S3B, C**). Analyses of the top ten GO terms most robustly associated with MEblack revealed altered synapse function as the most prominently altered biological function (**Figure S3E**). However, when we repeated the same analysis for female mice, we again found a single module (MEblue) with an eigengene stress x genotype interaction (**Figure S3G–I**), and the top ten GO terms again pointed to synapses as the most prominently altered function. (**Figure S3K**). Crucially, the eigengene interactions between stress and genotype in these modules were largely driven by increased eigengene values in the NS-resilient mice, and upregulation of these modules did not attenuate or reverse stress effects in the vulnerable mice. Moreover, the CVS-induced FC in expression of genes represented by the MEblack (male) and MEblue (female) modules were similarly anticorrelated between SSTCre and SSTCre:γ2^f/f^ mice (**Figure S3D, J**). Therefore, the GSEA-based NES data (**Figure S2G**) and WGCNA data (**Figure S3**) are consistent in that altered synapse function failed the litmus test of being male-specific and therefore could not contribute to the male-specific resilience mechanism in mPFC SST neurons (additional reasons against a role for altered synapse function are discussed).

## DISCUSSION

We have presented a detailed transcriptomic analysis of SST neurons in the mPFC associated with behavioral stress resilience of mice, driven by disinhibition of SST neurons. We found that SST neurons purified from the mPFC of male, but not female resilient mice are resilient to CVS-induced gene expression changes, as well as resilient to multiple CVS-responsive gene sets a priori associated with stress vulnerability. We previously showed that chemogenetic, chronic activation of SST neurons in the mPFC of male but not female mice is sufficient to induce resilience to chronic stress-induced changes in anxiety- and depression-related motivated behaviors ^41^ and tissue-level transcriptional changes ^40^. Altogether, the data indicate that chronic activation of SST neurons leads to stress resilience at the cellular level and that these properties of SST cells then drive resilience at the mPFC bulk transcriptome and behavioral levels ^40^.

More specifically, we observed that SST neurons from the mPFC of male SSTCre:γ2^f/f^ mice replicated multiple features of the corresponding tissue-level transcriptome ^40^. First, we observed fewer CVS-responsive DEGs in SST neurons from the resilient (SSTCre:γ2^f/f^) than from the vulnerable (SSTCre) male mice. Second, we found that the CVS-induced DEGs from SST neurons of vulnerable (SSTCre) male mice were strongly correlated with the corresponding genotype (SSTCre:γ2^f/f^ vs. SSTCre)-induced changes in NS mice. Third, we observed that the genotype-induced changes in NS SSTCre:γ2^f/f^ vs. SSTCre mice were partially reversed in CVS-exposed SSTCre:γ2^f/f^ mice. Lastly, all these features were observed specifically in male but not female mice, consistent with all the evidence that the mPFC represents a male-specific substrate for stress resilience. This sex difference is remarkable because the genetic ablation of γ2-GABA_A_ receptors leading to resilience in SSTCre:γ2^f/f^ mice is identical in male and female mice.

The observation that the CVS-induced transcriptome signature in SST neurons of vulnerable (SSTCre) male mice resembled the transcriptome signature of NS stress-resilient mice (e. g., represented by the SSTCre:γ2^f/f^ vs. SSTCre contrast) indicates that the stress-induced transcriptome of vulnerable mice includes a significant number of transcripts, which, when considered in isolation, can contribute to resilience. This interpretation is consistent with unexpected but well-supported findings that limited stress inoculation can promote resilience, both in humans ^49^ and mice ^46–48^.

Our data from SST neurons purified from the mPFC revealed marked sex differences in gene expression induced by chronic stress. These data extend findings by others who reported prominent sex differences in the basal transcriptomes of SST neurons purified from unstressed mice ^45^, the mPFC whole-tissue stress-induced transcriptomes of mice ^78^, and postmortem brain tissue from patients with MDD or PTSD ^6, 78^. In particular, we found that resilience in the male resilient (SSTCre:γ2^f/f^) mice was reflected in fewer stress-induced transcriptional changes in SST neurons than in their male vulnerable (SSTCre) littermates. By contrast, SST cells in the female resilient mice showed increased stress-induced DEGs compared to their vulnerable female littermates. This observation aligns with our prior tissue-level transcriptome ^40^ and chemogenetic ^41^ studies, demonstrating that SST neurons of the mPFC drive behavioral- and transcriptome-level resilience in male but not female mice. This sex difference appears to reflect the much greater baseline excitability of prefrontal SST neurons in male vs. female mice (Supplementary Figure 6M-O in Ref. ^68^). That is, the increased baseline excitability of SST cells in male vs. female mice is likely to result in a smaller relative increase in excitability in males than in females when excitability is further increased by ablation of GABA_A_ receptors. Nevertheless, this same study ^68^ showed that a one-hour immobility stressor significantly increased the excitability of SST neurons in male mice but not female mice, suggesting that the stress-induced increase in excitability observed in SST neurons of males is extrinsic to SST neurons. In keeping with this notion, PCA of RNA-Seq data from purified SST neurons resulted in the co-clustering of male and female data (**Figure S1H)**, whereas the sexes were clearly separated in our tissue-level transcriptomes ^40^. The collective evidence suggests that SST neurons, as well as other cell types (most likely Pvalb-positive interneurons ^79^) contribute to the prominent sex differences in CVS-induced prefrontal bulk tissue RNASeq data.

Our transcriptome data showed consistently that the SST-neuron-derived transcriptome of SSTCre female mice was less responsive to CVS than that of males. This is consistent with the above-mentioned sex differences in the response of SST cells to an acute stressor ^68^ and with the apical dendrites of pyramidal cells (the principal targets of SST neurons) in the mPFC being less vulnerable to stress in females than in males ^80–83^. Female vulnerable (SSTCre) mice showed fewer CVS-responsive DEGs than the males and largely unaltered expression of gene sets implicated in vulnerability. Moreover, the SST cell transcriptome of the female resilient (SSTCre:γ2^f/f^) mice differed from that of the males in that it had become more (not less) responsive to CVS than SST cells from their vulnerable SSTCre littermates, suggesting detrimental rather than pro-resilient consequences of SST neuron disinhibition.

Strikingly, the transcriptomic CVS response in SST neurons in female SSTCre:γ2^f/f^ mice was robustly anticorrelated with that in SSTCre controls, whereas no such opposite stress effects were observed in male mice. The behavioral relevance of these transcriptome changes remains elusive, as increasing SST neuron activity in the PLC of female mice did not affect behavior, independent of stress ^41^. However, known sex differences in the excitability of SST neurons provide a possible explanation for the anticorrelated stress-induced transcriptomes in the female mice. SST neurons in the PLC of female SSTCre mice exhibit a significantly lower baseline excitability than in males, and they lack the prominent increase in excitability observed after a 1-h immobility stressor, in contrast to males ^68^. It seems, therefore, that SST neurons in female SSTCre mice are effectively protected from the detrimental consequences of stress exposure, most likely due to stress-induced feedback inhibition mediated by Pvalb interneurons. In support of this explanation, chronic stress results in a prominent and lasting increase in Pvalb neuron excitability in female but not male mice ^84^. The absence of postsynaptic GABA_A_ receptors in SST neurons in SSTCre:γ2^f/f^ mice ^30^ is predicted to prevent this stress-induced feedback inhibition and to result in a stress-induced net increase of excitatory drive to SST cells. Therefore, while stress of SSTCre female mice increases the network-mediated inhibitory drive to SST neurons, stress of SSTCre:γ2^f/f^ female mice results in a switch from primarily inhibitory network effects via tonic inhibition (which is maintained in SSTCre:γ2^f/f^ vs. SSTCre mice ^30^) to an excess in network-mediated excitation of SST neurons. This switch in the network-mediated effect of stress explains the opposite transcriptome responses in the two strains of mice. By contrast, in male mice, stress exposure increases excitatory drive to SST neurons in both SSTCre mice ^68^ and SSTCre:γ2^f/f^ mice, with a likely much larger increase in the latter, due to the increased input resistance and absence of feedback inhibition ^30^.

GSEA-based analyses of GO summary terms indicated that stress resilience of the male mice involves attenuation of CVS-induced downregulation of neuronal cell fate, apoptosis/PCD, cell adhesion, MAPK pathways, and the cytoskeleton. All of these functions were measurably down-regulated in vulnerable male but not female mice, which aligns with the premise that the mechanism underlying stress resilience is male-specific. Two of these functional domains (apoptosis/PCD, cell adhesion) showed comparable downregulation in CVS vs. NS SSTCre (vulnerable) male mice and in NS SSTCre:γ2^f/f^ (resilient) vs. NS SSTCre (vulnerable) male mice, which aligns with the strong transcriptional concordance we observed between these two contrasts, indicating that SST neurons of NS resilient mice mimic aspects of stress-exposed vulnerable mice and replicate corresponding findings from our previous analyses of the mPFC tissue-level transcriptomes ^40^. Importantly, while the SST cell transcriptome of NS resilient mice resembles that of SST neurons from stress-exposed vulnerable mice, we had shown that the resilient mice showed no behavioral or hormonal signs of stress axis activation and therefore do not experience a systemic stress response ^40^.

The MAPK pathway- and cytoskeleton-related transcripts were similarly downregulated by stress in the vulnerable and resilient male mice, suggesting a contribution of natural stress-coping responses to resilience. We have previously described putative natural stress-coping genes in our tissue-level analysis of resilience-related transcriptomes ^40^.

Our data are consistent with opposite transcriptional signatures of depression in men and women ^85^ and previously reported sex-specific effects of CVS on the bulk transcriptomes of stress-vulnerable wildtype mice ^78^. Similar to others ^35, 64, 86^, we found evidence of CVS-induced downregulation of mRNA translation in SST neurons from vulnerable mice (**Figure S3F)**. However, except for “heat“ and “response to heat”, none of the GO terms related to ER stress or the UPR were significantly enriched. Therefore, we could not corroborate the finding that downregulation of translation in SST neurons involved activation of the ER stress/UPR pathway (**Figure S3H**). Moreover, while others reported chronic stress-induced upregulation of synaptic adhesion-related genes in SST neurons ^86^, we found evidence for prominent stress-induced downregulation of corresponding GO summary terms, and these effects were male-specific and normalized in SST neurons from CVS-exposed stress-resilient mice, strongly suggesting that they were relevant to the stress-resilient phenotype of the male SSTCre:γ2^f/f^ mice. Possible sources for discrepancies between studies include the mouse strains used (wildtype vs. SSTCre:tdT transgenic), the stress protocols used (UCMS vs CVS), the time point after the last stressor at which the tissue was harvested (12 vs. 24–28 h), and differences in the methods used to enrich for SST neurons (in situ hybridization/laser capture microdissection of fixed tissue vs. FACS purification of live neurons). Interestingly, the translation-related GO terms were downregulated by CVS not only in the vulnerable (SSTCre) mice but also in the resilient (SSTCre:γ2^f/f^) mice (**Figure S3F).** This CVS effect in the resilient mice is opposite to the upregulation we had observed for the corresponding tissue level transcriptome ^40^, suggesting that the CVS-induced increase in mRNA translation associated with resilience maps to cell types other than SST neurons. Similarly, the apoptosis and programmed cell death-related terms were downregulated by CVS in the resilient mice, which is opposite to the stress effect observed in vulnerable mice described by others at the cortical tissue level ^71^, and suggests that the CVS effects on apoptosis in SST interneurons are opposite to those in other cell types.

In an attempt to corroborate findings on the resilience-related biological functions of SST neurons based on GSEA and NES of GO terms, we analyzed WGCNA-based gene modules and GO terms. However, these experiments were not informative, most likely because WGCNA was underpowered. The only gene module that showed an interaction of stress and genotype effects in male mice pointed to enhanced synapse function as a resilience mechanism. However, a very similar module was detected in females, which disqualified this module from being relevant to the male-specific resilience mechanism. This holds even though the gene networks underlying these modules were distinct in male and female mice, most likely due to the sex difference in basal excitability of SST neurons ^68^. Most decisively, our previous functional characterization of SSTCre:γ2^f/f^ mice had confirmed that inhibitory synapses recorded from SST neurons were drastically impaired as designed and illustrated by the > 50% reduction in the frequency and amplitude of postsynaptic currents. This was expected due to the ablation of γ2-GABA_A_ receptors in these neurons, while glutamatergic synapses and tonic inhibition remained unchanged ^30^. It follows that upregulation of synapse-related gene networks in SST neurons from SSTCre:γ2^f/f^ mice represents a functionally inconsequential transcriptional response to the deletion of an essential subunit of GABA_A_ receptors ^87^.

The loss and maintenance of SST neurons has recently been tied to vulnerability and resilience during normal aging and in subjects with Alzheimer’s neuropathology ^33, 88–90^. Conversely, aging-related cognitive decline is comorbid with depressive disorder, indicating overlapping disease etiologies ^5, 91, 92^. Future work will need to examine whether increasing the excitability of SST neurons confers resilience to cognitive decline in Alzheimer’s disease, and whether such a link might be governed by a sex-specific substrate similar to that identified here for stress resilience.

## Supplementary Material

This is a list of supplementary files associated with this preprint. Click to download.

- 20260809ShaoetalSupplement.docx

## Figures and Tables

**Figure 1.**
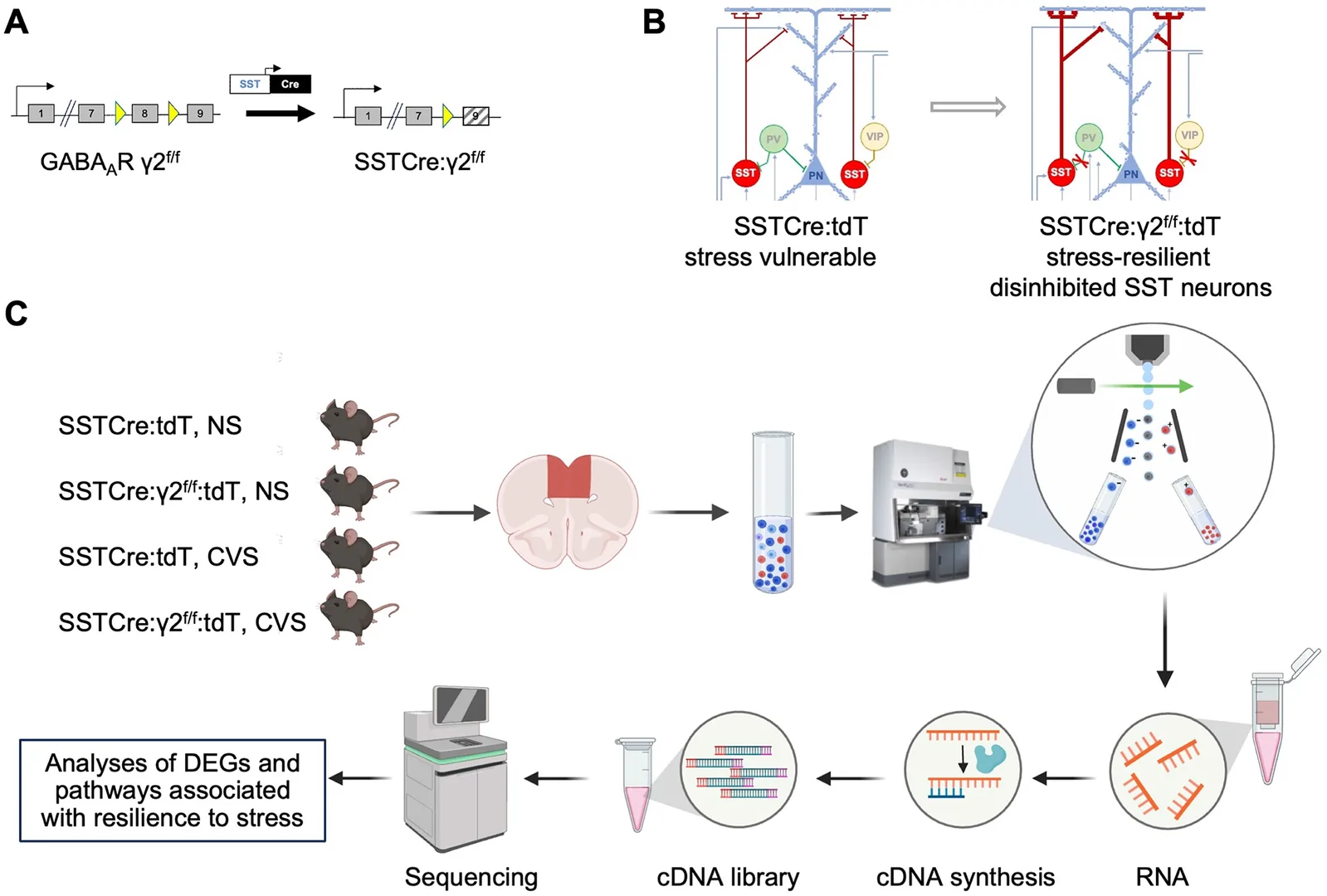
Experimental design. **A**) SSTCre: γ2^f/f^ mice were generated by crossing SSTCre mice with GABA_A_ receptor γ2^f/f^ mice carrying a floxed allele of the *Gabrg2* gene, as previously described ^30^. **B**) SSTCre-induced disinhibition of SST neurons. Inactivation of the *Gabrg2* gene in SST neurons results in a drastic reduction of GABAergic inhibitory synaptic input from Pvalb- and VIP-positive interneurons, along with a significant increase in the input resistance and intrinsic excitability of SST neurons, which in turn increases the frequency and amplitude of inhibitory synaptic inputs to the distal dendrites of pyramidal cells ^30^. Behaviorally, these mice mimic the effect of antidepressant drug treatment ^30^ along with stress resilience, independent of sex ^40^. **C**) SSTCre:tdT and SSTCre:γ2^f/f^:tdT littermate mice were separated by sex and genotype. Equal groups of mice were subjected to CVS or NS control conditions. The mPFC was dissected, dissociated, stained with calcein, followed by FACS of tdT^+^/calcein^+^ neurons, RNA isolation, cDNA synthesis, library construction, RNA sequencing, and analyses of DEGs and whole-genome transcriptomes.

**Figure 2.**
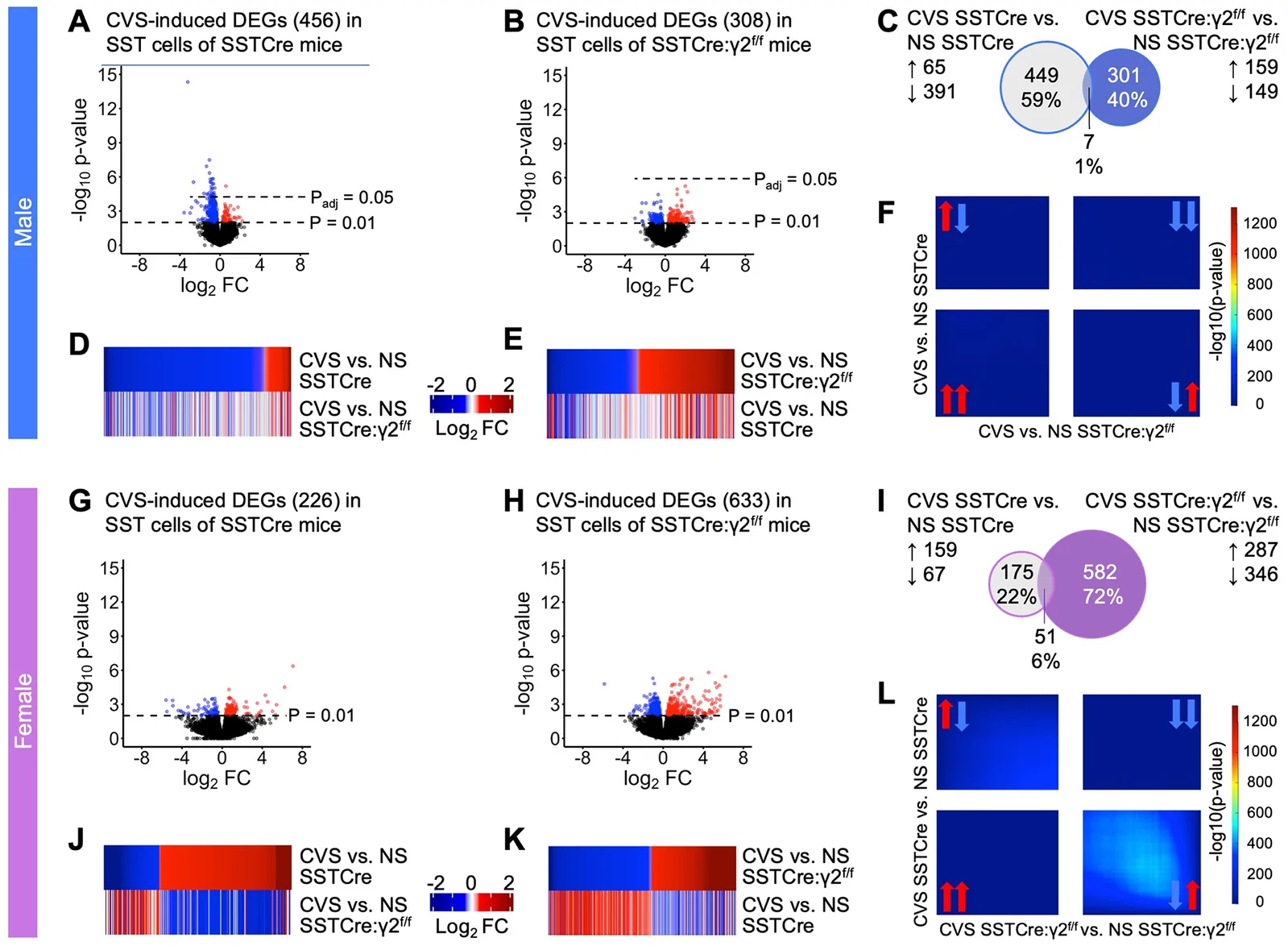
CVS-induced DEGs in SST neurons purified from the mPFC of male but not female stress-resilient (SSTCre:γ2^f/f^) mice are fewer and distinct from those in SSTCre controls. **A, B**) Volcano plot of CVS-induced DEGs (p < 0.01) in SST neurons purified from the mPFC of SSTCre mice (vulnerable, A) and SSTCre:γ2^f/f^ (male mice, resilient, B) (n = 4). The position of the dashed line indicating P_adj_ = 0.05 is approximate and without a proper scale. **C**) Venn diagram comparing the CVS-induced DEGs of the two strains of mice. Note the lower nominal number of DEGs in the resilient vs. vulnerable mice, confirming the data in **Figure S2**. **D**) Heat maps comparing the CVS-induced DEGs detected in SST neurons from SSTCre mice (log_2_FC, top) to the transcriptional changes of the same genes in SST neurons of SSTCre:γ2^f/f^ mice (bottom). **E**) Heat maps comparing the directional fold changes of CVS-responsive DEGs detected in SST cells in SSTCre:γ2^f/f^ mice (top) with the corresponding genes in SSTCre controls (bottom). Note that the majority of DEGs are downregulated in the SSTCre (vulnerable) mice (top row in D) and more evenly distributed in the SSTCre:γ2^f/f^ (resilient) mice (top row in (E). The directional changes of DEGs (top rows in D, E) show random directional changes of the corresponding genes in the comparison strain (bottom rows in D, E). **F**) RRHO plots of CVS-induced transcriptomes in SST neurons from SSTCre mice compared to SSTCre:γ2^f/f^ mice. Heat indicates the strength of overlap, and quadrants represent the direction of gene expression (lower-left quadrant, genes upregulated in both conditions; upper-right quadrant, genes downregulated in both conditions; upper left quadrant, genes up in SSTCre:γ2^f/f^ and down in SSTCre mice; lower right quadrant: genes down in SSTCre:γ2^f/f^ and up in SSTCre mice. Note the absence of concordant and discordant expression in males, which confirms the lack of concordant and discordant DEGs in (D, E). **G-L**) Data as in (A-F) but for female mice. Note the increased number of CVS-induced DEGs in SSTCre:γ2^f/f^ vs. SSTCre mice in (G-I), which is opposite to the data in males. Heat maps (J) indicate that CVS-responsive DEGs in female SSTCre mice are preferentially upregulated, which is again different from the males (D). DEGs detected in SSTCre (J, upper row) and SSTCre:γ2^f/f^ female mice (K, upper row) show opposite directional changes of the same transcripts in the other strain (bottom rows)(J, K). RRHO plots of CVS-responsive transcripts confirm the significant discordant CVS effects in SSTCre vs. SSTCre:γ2^f/f^ female mice (L).

**Figure 3.**
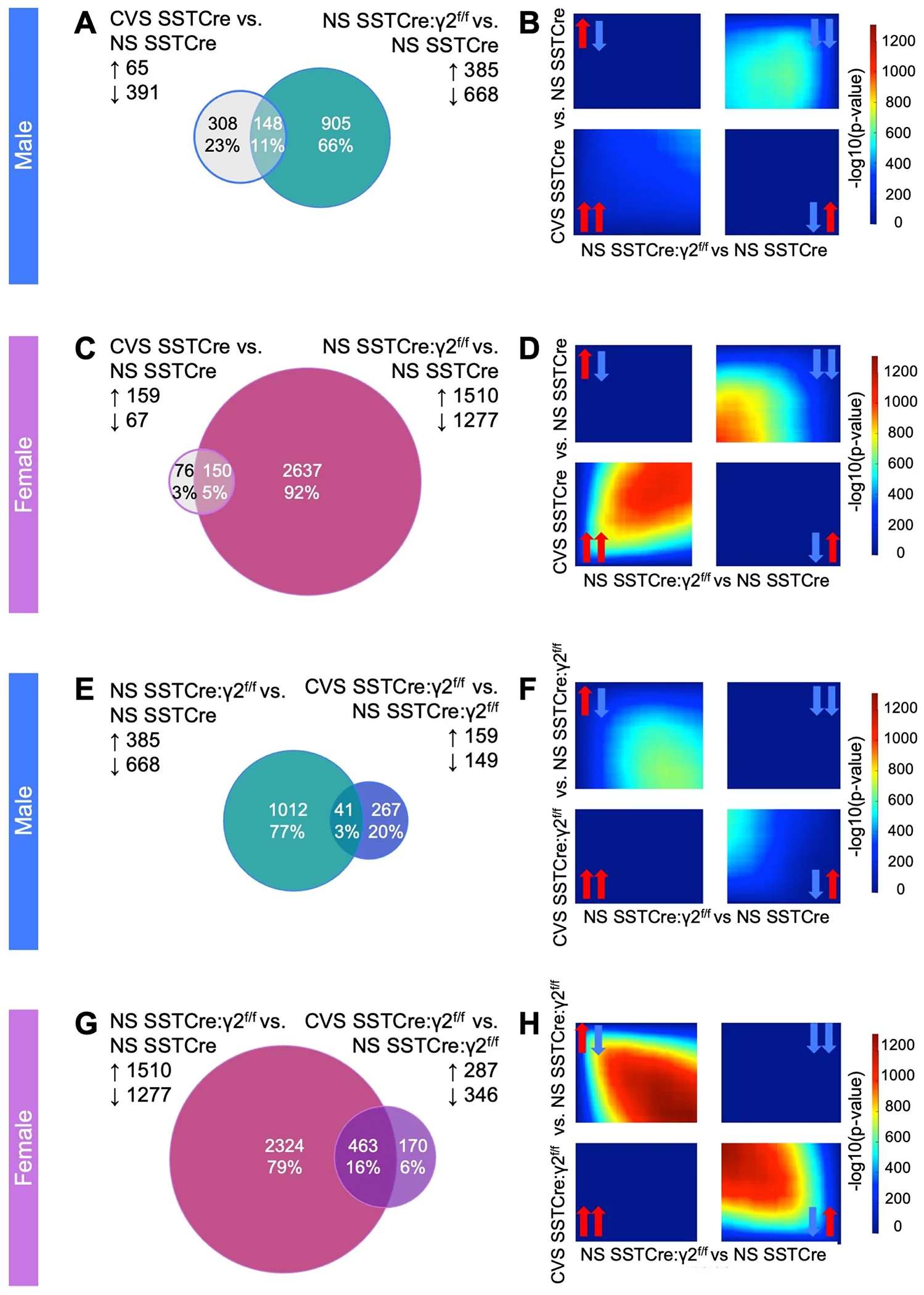
Comparison of CVS-induced DEGs with genotype-induced DEGs. **A**) Venn diagram comparing the CVS-induced DEGs in SST cells from SSTCre mice with the genotype-induced DEGs in NS male mice (NS SSTCre vs NS SSTCre:γ2^f/f^). **B**) RRHO analyses of the same contrast. Note the significant concordant gene expression changes that are especially prominent for transcripts that are downregulated under the two conditions. **C, D**) Data as in (A,B) but for female mice. Note the much greater number of DEGs induced by disinhibition of SST neurons in SSTCre mice than by CVS exposure of SSTCre mice. RRHO analyses show prominent concordant effects of CVS and disinhibition, which are most striking for transcripts upregulated under both conditions. **E**) Venn diagram comparing the genotype effect to the effect of CVS on stress-resilient male mice. Note the greater effect of SST neuron disinhibition compared to CVS exposure of stress-resilient mice. **F**) RRHO analyses revealed prominent discordant effects, indicating that genes that were upregulated in SST neurons by disinhibition of SST neurons were downregulated (or normalized) by stress exposure of the stress-resilient mice, and vice versa. **G, H**) Data as in (E, F) but for female mice. Note the discordant effects of the two contrasts, similar to but more prominent than those observed in males.

**Figure 4.**
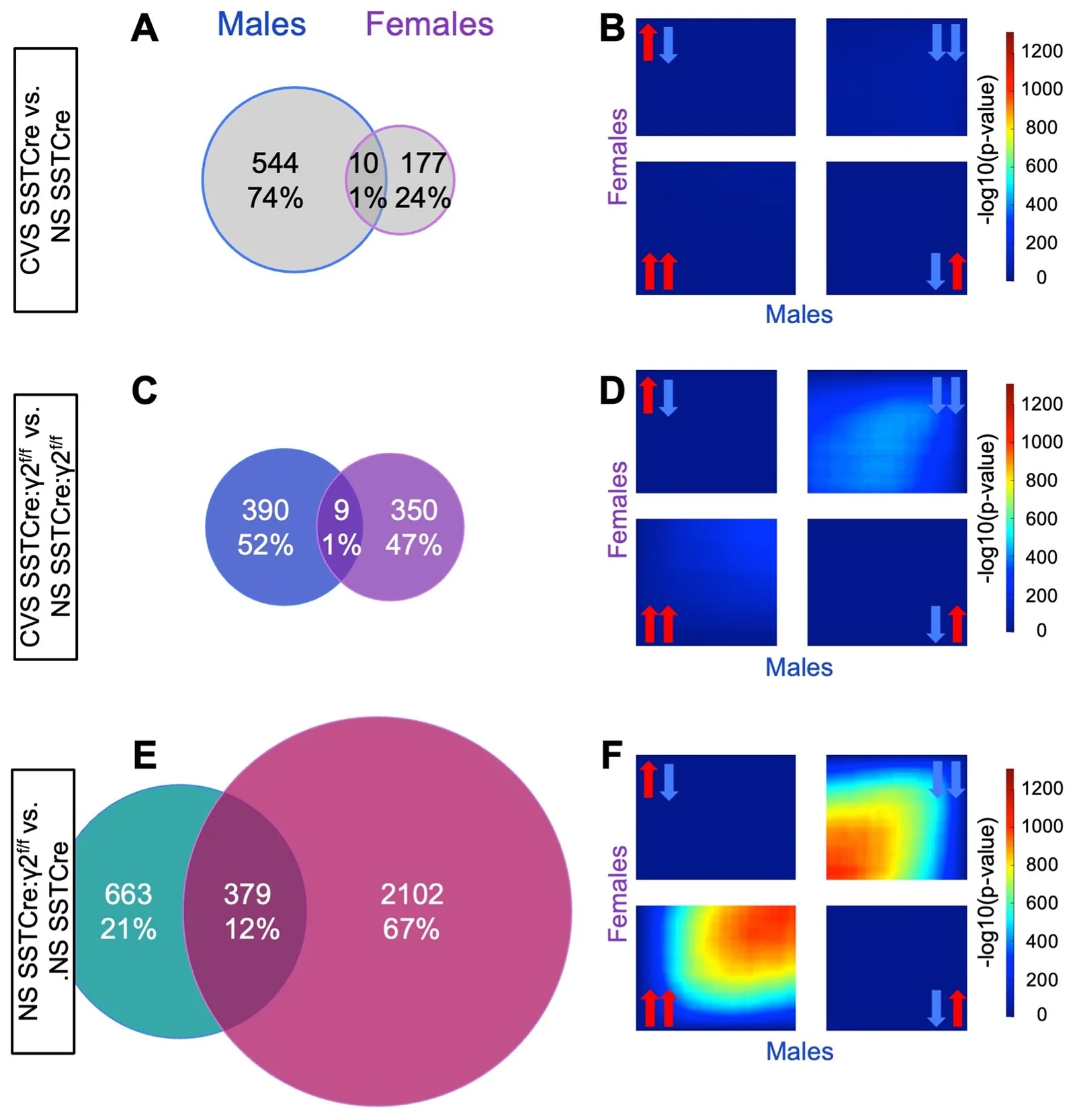
Comparison of CVS- and genotype-induced DEGs between male and female mice. **A, B**) Venn diagram comparing CVS-induced DEGs (A) and the CVS-induced transcriptomes changes in SST neurons from male vs. female SSTCre mice (B). Note the negligible overlap of DEGs and the absence of significant concordant and discordant CVS effects, indicating that the CVS responses in SST neurons of male and female SSTCre mice are distinct. **C, D**) Venn diagram comparing CVS-induced DEGs (C) and the CVS-induced transcriptomes changes in SST neurons from male vs. female SSTCre:γ2^f/f^ mice (D). Note the significant concordance between the CVS-induced transcriptomes of male and female SSTCre:γ2^f/f^ mice, especially for DEGs downregulated under both conditions. **E, F**) Venn diagram comparing genotype-induced DEGs (E) and the effect of disinhibition of SST neurons in NS male vs female mice (F). Note the robust concordant effects of disinhibition on the transcriptomes of SST neurons in male vs. female mice, similarly evident for upregulated and downregulated genes.

**Figure 5.**
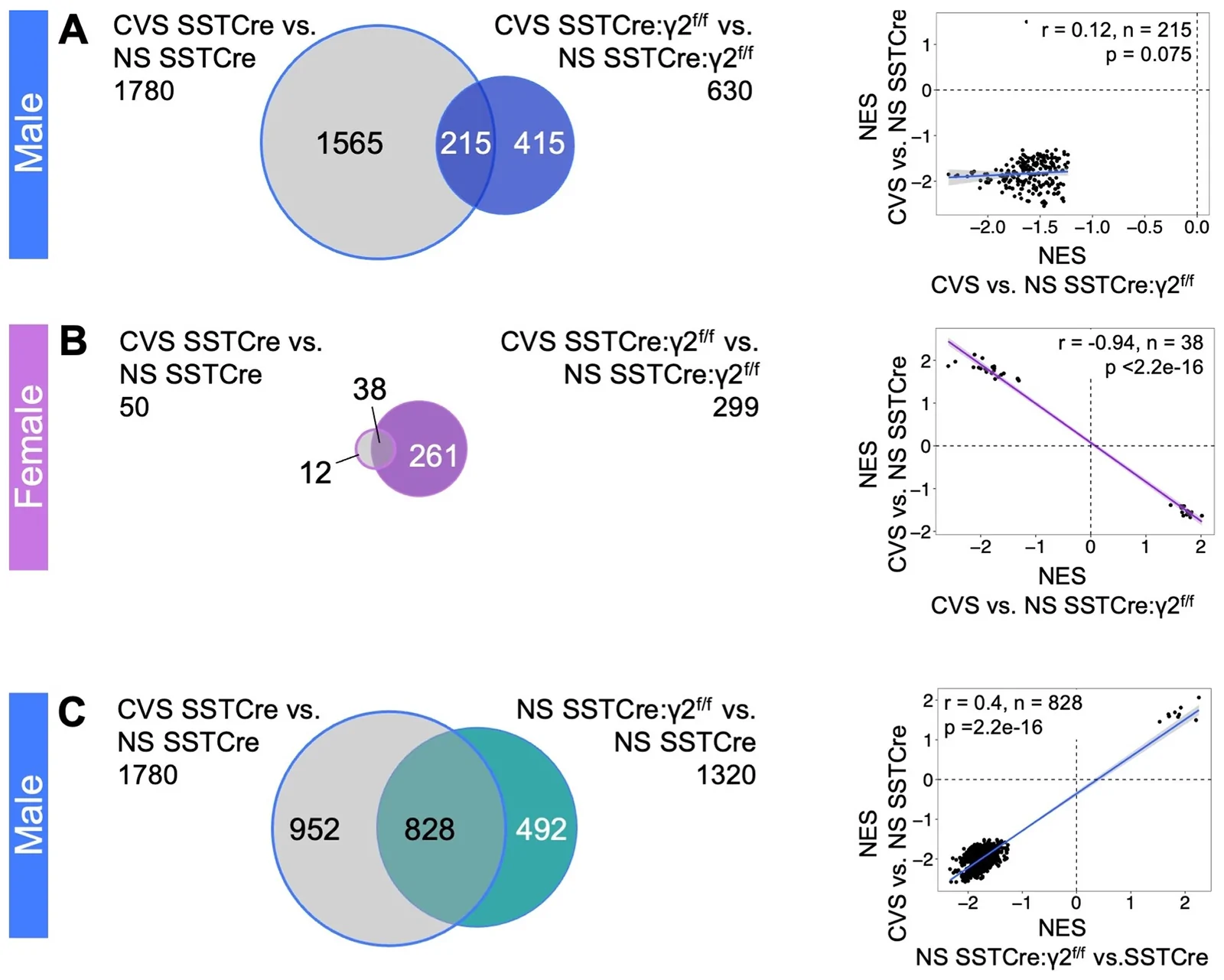
Comparison of CVS and genotype-responsive gene sets by GSEA and NES. **A)** Venn diagram of the CVS-induced gene sets determined by GSEA (p_adj_ < 0.05), comparing SST cells from male SSTCre vs. SSTCre:γ2^f/f^ mice, and correlational analyses in NES of overlapping gene sets. **B**) The same analyses as in (A) but from female mice. Note the lack of correlation in NES for overlapping gene sets in SST cells from male mice and the strong negative correlation in females. **C**) Comparison of the CVS-induced gene sets in SST cells from male SSTCre mice with the genotype-induced gene sets in SST cells from male NS SSTCre vs. NS SSTCre:γ2^f/f^ mice, by Venn diagram of GSEA and correlational analysis of NES of overlapping gene sets. Note the large overlap of the CVS- and genotype-induced gene sets and the positive correlation of the corresponding NES.

**Table 1:** Summary of GSEA terms differentially affected by CVS and stress resilience. Based on the NES values, the biological summary terms implicated in resilience were grouped into ↓↓↓ strongly downregulated, ↓↓ downregulated, ↓ modestly downregulated, ↔ unchanged, ↑↑↑ strongly upregulated, ↑↑ upregulated, and ↑ modestly upregulated. nd, gene set was not detected/not affected. Biological functions depicted in bold show stress effects in SSTCre male mice that are fully or partially attenuated in SSTCre:γ2^f/f^ male mice but not in females, suggesting they contribute to the stress-resilience-like properties of SST neurons in the mPFC of SSTCre:γ2^f/f^ male mice. Note that synapse-related GO terms were unaffected by stress in vulnerable mice of both sexes, arguing against a role for synapse-related functions in SST neurons from stress resilience of SSTCre:γ2^f/f^ male mice. Similar changes in synapse-related functions in male and female mice further preclude a biologically meaningful contribution to the sex-specific resilience of male SSTCre:γ2^f/f^ mice.

GSEA Summary GO terms differentially regulated by CVS in stress-vulnerable vs. stress-resilient male mice, and compared to genotype effects	♂CVS SSTCre vs. NS SSTCre stress effect on stress-vulnerable mice	♂CVS SSTCre:γ2^f/f^ vs. NS SSTCre:γ2^f/f^ stress effect on stress-resilient mice	♂NS SSTCre:γ2^f/f^ vs. NS SSTCre stress resilient vs stress-vulnerable non-stressed mice	♀ CVS SSTCre vs. NS SSTCre stress effect on stress-vulnerable mice	♀ CVS SSTCre:γ2^f/f^ vs. NS SSTCre:γ2^f/f^ stress effect on stress-resilience mice	♀ NS SSTCre:γ2^f/f^ vs. NS SSTCre stress resilient vs stress-vulnerable non-stressed mice
**Neuronal Cell Fate**	↓	↔	↔	nd	nd	nd
**Apoptosis, PCD**	↓↓	↔	↓	↔	↔	↔
**Cell Adhesion**	↓↓↓	↔	↓↓	↔	↑	↓
**MAPK Pathway**	↓↓	↓	↔	↔	↔	↓
**Cytoskeleton**	↓↓	↓	↔	↔	↔	↓
Translation	↓	↓↓	↔	↔	↓	↑↑
Synapse	↔	↓↓↓	↑↑↑	↔	↓↓	↑↑↑
ER Stress/UPR	nd	nd	nd	nd	nd	nd
